# Case report: An asymptomatic mother with an inborn error of cobalamin metabolism (cblC) detected through high homocysteine levels during prenatal diagnosis

**DOI:** 10.3389/fnut.2023.1124387

**Published:** 2023-05-12

**Authors:** Yu-Peng Liu, Ru-Xuan He, Zhe-Hui Chen, Lu-Lu Kang, Jin-Qing Song, Yi Liu, Chun-Yan Shi, Jun-Ya Chen, Hui Dong, Yao Zhang, Meng-Qiu Li, Ying Jin, Jiong Qin, Yan-Ling Yang

**Affiliations:** ^1^Department of Pediatrics, Peking University People's Hospital, Beijing, China; ^2^Department of Pediatrics, Peking University First Hospital, Beijing, China; ^3^Department of Respiratory, Beijing Children′s Hospital, Capital Medical University, Beijing, China; ^4^Department of Pediatrics, The First Affiliated Hospital of Zhengzhou University, Zhengzhou, China; ^5^Department of Clinical Laboratory, China-Japan Friendship Hospital, Beijing, China; ^6^Department of Gynaecology and Obstetrics, Peking University First Hospital, Beijing, China

**Keywords:** methylmalonic acidemia, homocysteinemia, asymptomatic, cblC type, prenatal diagnosis

## Abstract

**Background:**

The most common disorder of the intracellular cobalamin metabolism pathway is the combined methylmalonic acidemia and homocysteinemia, cblC type (cblC). There is a variation in its clinical spectrum ranging from severe neonatal-onset forms that are highly fatal to later-onset forms which are milder. In this study, the first case of an asymptomatic Chinese woman with a defect in congenital cobalamin (cblC type) metabolism at prenatal diagnosis due to elevated homocysteine level is identified.

**Case presentation:**

The proband, a male child born to a 29-year-old G1P0 mother, admitted to local hospital with feeding disorder, intellectual disability, seizures, microcephaly, as well as heterophthalmos. The level of the urine methylmalonic was elevated. Equally found were increased blood propionylcarnitine (C3) and propionylcarnitine/free carnitine ratio (C3/C0) and decreased methionine levels. The plasma total homocysteine level was elevated at 101.04 μmol/L (normal < 15 μmol/L). The clinical diagnosis of combined methylmalonic acidemia and homocysteinemia was supported. Four years later, the mother of the boy married again and came to us for prenatal diagnosis exactly 15 weeks after her last menstrual period. Subsequently, there is an increase in the amniotic fluid methylmalonate. The level of the amniotic fluid total homocysteine was marginally high. A considerably elevated amniotic fluid C3 was equally observed. In addition, there is a respective significant increase in the plasma and urine total homocysteine at 31.96 and 39.35 μmol/L. After the sequencing of MMACHC genes, it is found that the boy, a proband carried a homozygous mutation of the *MMACHC* at c.658_660delAAG. While the boy's mother, she carries two mutations in *MMACHC*: c.658_660delAAG and c.617G>A. The fetus is a carrier of the *MMACHC* gene. Following the administration of routine treatment, the mother remained symptom-free in the course of pregnancy, and she gave birth to a healthy boy.

**Conclusion:**

Variable and nonspecific symptoms characterized the cblC type of methylmalonic acidemia combined with homocysteinemia. Both biochemical assays and mutation analysis are recommended as crucial complementary techniques.

## Introduction

Methylmalonic aciduria (MMA), a rare inherited disorder, comprise a group of genetically heterogeneous autosomal recessive disorders caused by defective metabolic pathways involving methylmalonyl-CoA mutase (MUT) or its cofactor, cobalamin (1). Combined methylmalonic acidemia and homocysteinemia, cblC type (cblC; OMIM #609831, #277400), denoted a consistent disorder of the intracellular cobalamin metabolism pathway, is the most common organic aciduria in China (2, 3). From prenatal detection to adulthood, there may be variation in severity in the clinical manifestation of cblC in affected patients ranging from mild to life-threatening (3–5). The relatives of these patients are burdened with heavy financial burdens. An increasing hospitalization with various presentations and a heavy financial burden per hospitalization were observed in Mainland China, while the medical resources were still relatively centralized in some districts, such as Beijing, Shanghai and Guangzhou (6). Therefore, crucial information could be provided through prenatal diagnosis concerning a decision about pregnancy that involves a fetus suffering from cblC defect that tends to subsequently minimize the social and family pressure brought by this defect (3). Reports from the literature have shown that during prenatal diagnosis of methylmalonic acidemia, prompt reliable results could be provided utilizing biochemical analysis of amniotic fluid (7–9). The challenge posed by the absence of genetic assessment for families prone to cblC defect fetuses could be resolved by applying these techniques (8, 10). Thus, this study reports the case of an asymptomatic pregnant mother who suffered from a congenital anomaly of cobalamin metabolism (cblC type) during prenatal diagnosis.

## Case presentation

The proband, a male child was a full-term infant born to a 29-year-old G1P0 mother, through the process of normal spontaneous vaginal delivery following a pregnancy that was uneventful. The family history did not indicate an occasion/occurrence of metabolic diseases. His admission into the local hospital was due to the diagnosis of disorders in the boy such as feeding disorder, intellectual disability, seizures, microcephaly, as well as heterophthalmos when he was a year old. His brain MRI result showed diffuse cerebral atrophy. Furthermore, the EEG was equally abnormal in the absence of dominant rhythm in the bilateral occipital area. The level of the urine methylmalonic was 516.44 mmol/mol creatinine (normal 0.2–3.6 mmol/mol creatinine). Equally found were increased blood propionylcarnitine (C3) and propionylcarnitine/free carnitine ratio (C3/C0) and decreased methionine levels. The plasma total homocysteine level was elevated at 101.04 μmol/L (normal <15 μmol/L). The clinical diagnosis of combined methylmalonic acidemia and homocysteinemia was supported through the biochemical findings. Regrettably, once the diagnosis was conducted, he failed to receive standardized treatment.

**Figure 1:**
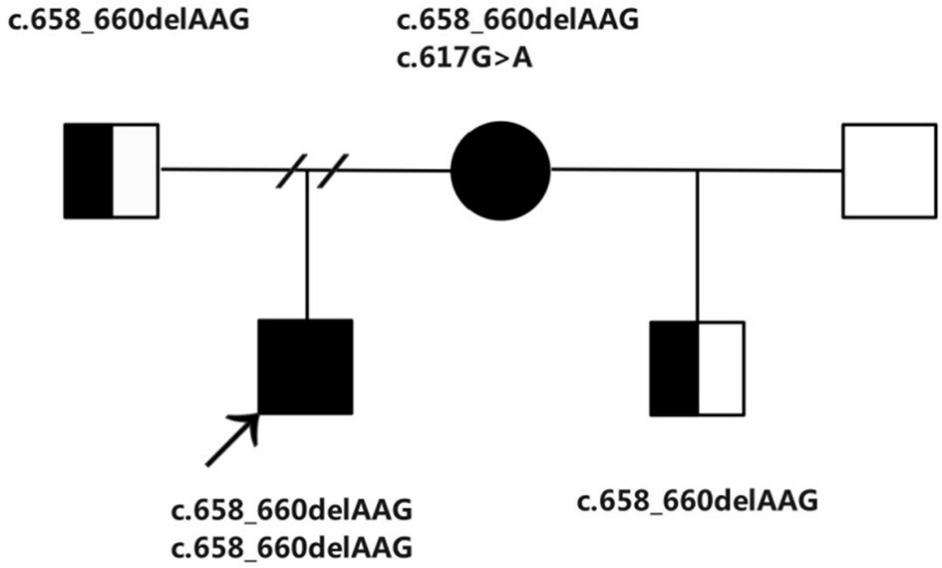
The family pedigree showing the mutations detected in MMACHC.

Moreover, after 4 years, the mother of the boy married again and was pregnant at age 33. She came to us for genetic counseling and prenatal diagnosis exactly 15 weeks after her last menstrual period. Subsequently, she was subjected to medical check-ups and metabolic assessments. The amniotic fluid sample was conducted in our hospital at the Department of Obstetrics and Gynecology. There is an increase in the GC–MS detected amniotic fluid methylmalonate to 3.08 mmol/mol creatinine (normal < 0.2 mmol/mol creatinine). At 6.06 μmol/L (normal < 5 μmol/L), the level of the amniotic fluid total homocysteine was marginally high. Furthermore, a considerably elevated amniotic fluid C3 at 5.13 μmol/L (normal < 3 μmol/L) was equally observed by the metabolic workup assessed through the LC–MS/MS (11–14). In addition, there is a respective significant increase in the plasma and urine total homocysteine at 31.96 and 39.35 μmol/L. Is this fetus assessed as “affected”? Or can we assume that the real patient is the mother? Sequencing of genes related to cobalamin metabolism has been performed for this family. From the research outcome, it is found that the boy, a proband carried a homozygous mutation of the *MMACHC* at c.658_660delAAG(p.Lys220del). While in the case of the boy's mother, she carries two mutations in *MMACHC*: c.658_660delAAG(p.Lys220del) and c.617G>A(p.Arg206Gln). The fetus is a carrier of the *MMACHC* gene. Cobalamin C disorder (cblC) was both diagnosed in the boy and his mother (Figure 1). The fetus was unaffected as the boy's mother was an asymptomatic patient. Following the administration of routine treatment for the mother: cobalamin mixture or hydroxocobalamin (1 mg, twice per week, i.m.), calcium folinate (15 mg/day p.o.), L-carnitine (2 g/day p.o.) and betaine (2 g/day p.o.), as well as a normal diet (15–17), she remained symptom-free, and there were no neurological abnormalities found on clinical examination. The routine blood and biochemical tests were normal and the urine methylmalonate and plasma total homocysteine levels-maintained stability in the course of pregnancy. After a gestation period spanning 38 weeks, she went into labor and gave birth to a healthy boy.

## Discussion

CblC defects denote an inborn defect caused by intracellular cobalamin metabolism (2). According to our earlier assertion, this defect also represents congenital organic aciduria, which is most common in mainland China (3, 4). It has complicated clinical manifestations ranging from asymptomatic to deadly having an extensive range of manifesting from prenatal period to adulthood. Patients afflicted with this form of the disease are often diagnosed with episodes of chronic metabolic acidosis, hypotension, developmental delay, encephalopathy and even death (3, 4, 18). Regarding patients with mild forms, they tend to have an asymptomatic period and only manifest later at school age or adulthood where symptoms that are manifested include psychomotor degeneration, seizures, the decline in school or work performance, social withdrawal, and neuropsychiatric disturbances (19–21). Till recently, the fact that an unaffected infant was born to a woman with a mild form, i.e., asymptomatic cblC diagnosed after a positive newborn screening (NBS) for low carnitine (22). And a case of pregnancy in a symptom-free patient with cobalamin C/D deficiency diagnosed through family screening (23).

Over the past 22 years, as advances are recorded in selective screening and neonatal screening, treatment with favorable outcomes has been achieved in an increasing number of Chinese patients afflicted with this disease, which has led to proper health care and care delivery for some of them when they reach childbearing age (3, 4). Nevertheless, the long-term outcomes are still not satisfactory, particularly in patients whereby the disease manifested at an early stage due to chronic neurological sequelae. The families of these patients bear significant financial and psychological burdens. Nevertheless, it is evident that biochemical techniques also have their shortcomings (8).

In order to determine if the fetus was “affected”, this study reported that the metabolite analysis of acylcarnitines by LC–MS/MS, organic acids by GC–MS, and total homocysteine in amniotic fluid were all elevated. Moreover, both urine and blood samples were collected from the pregnant woman. The result demonstrated that there is a significant increase in total homocysteine levels in blood and urine compared to amniotic fluid. The pregnant woman was suspected of having methylmalonic acidemia associated with homocysteinemia. From gene assessment, it is confirmed two mutations in *MMACHC* were carried by the mother. According to her diagnosis, she had a cobalamin C (cblC) disease. The fetus was a carrier of the MMACHC gene. The c.658_660delAAG and c.617G>A were all reported, and predicted to be damaging (24, 25). The c.658_660delAAG was one of the most common mutations in China (26). It seems that a rapid and precise technique for the prenatal diagnosis of methylmalonic acidemia is the biochemical analysis of amniotic fluid samples using GC–MS and/or LC–MS/MS combined with the total homocysteine assay; nevertheless, it is extremely recommended to incorporate the gene assessment during parental diagnosis (8).

Conclusively, variable and non-specific symptoms characterized the cblC type of methylmalonic acidemia combined with homocysteinemia. A reliable, albeit imperfect, biochemical method of prenatal diagnosis is the combination of total homocysteine assay with acyl-carnitine and organic acid assay. For the entire case, both biochemical assays and mutation analysis are recommended as crucial complementary techniques.

## Data availability statement

The original data presented in the study are included in the article, further inquiries can be directed to the corresponding authors.

## Ethics statement

The studies involving human participants were reviewed and approved by Peking University First Hospital. Written informed consent to participate in this study was provided by the participants' legal guardian/next of kin. Written informed consent was obtained from the individual(s), and minor(s)' legal guardian/next of kin, for the publication of any potentially identifiable images or data included in this case report.

## Author contributions

JQ and Y-LY designed the study. Y-PL drafted the manuscript and analyzed the data. Z-HC, L-LK, R-XH, YL, C-YS, J-YC, HD, and YZ participated in the clinical management and patient data collection. J-QS, M-QL, and YJ helped with the patient data collection. All authors contributed to the article and approved the submitted version.
